# Are Children Like Werewolves? Full Moon and Its Association with Sleep and Activity Behaviors in an International Sample of Children

**DOI:** 10.3389/fped.2016.00024

**Published:** 2016-03-24

**Authors:** Jean-Philippe Chaput, Madyson Weippert, Allana G. LeBlanc, Mads F. Hjorth, Kim F. Michaelsen, Peter T. Katzmarzyk, Mark S. Tremblay, Tiago V. Barreira, Stephanie T. Broyles, Mikael Fogelholm, Gang Hu, Rebecca Kuriyan, Anura Kurpad, Estelle V. Lambert, Carol Maher, Jose Maia, Victor Matsudo, Timothy Olds, Vincent Onywera, Olga L. Sarmiento, Martyn Standage, Catrine Tudor-Locke, Pei Zhao, Anders M. Sjödin

**Affiliations:** ^1^Children’s Hospital of Eastern Ontario Research Institute, Ottawa, ON, Canada; ^2^ParticipACTION, Toronto, ON, Canada; ^3^University of Copenhagen, Copenhagen, Denmark; ^4^Pennington Biomedical Research Center, Baton Rouge, LA, USA; ^5^University of Syracuse, Syracuse, NY, USA; ^6^University of Helsinki, Helsinki, Finland; ^7^St. Johns Research Institute, Bangalore, India; ^8^University of Cape Town, Cape Town, South Africa; ^9^University of South Australia, Adelaide, SA, Australia; ^10^University of Porto, Porto, Portugal; ^11^Centro de Estudos do Laboratório de Aptidão Física de São Caetano do Sul (CELAFISCS), Sao Paulo, Brazil; ^12^Kenyatta University, Nairobi, Kenya; ^13^Universidad de los Andes, Bogota, Colombia; ^14^University of Bath, Bath, UK; ^15^University of Massachusetts Amherst, Amherst, MA, USA; ^16^Tianjin Women’s and Children’s Health Center, Tianjin, China

**Keywords:** moon, lunar cycle, sleep, physical activity, sedentary behavior, children

## Abstract

In order to verify if the full moon is associated with sleep and activity behaviors, we used a 12-country study providing 33,710 24-h accelerometer recordings of sleep and activity. The present observational, cross-sectional study included 5812 children ages 9–11 years from study sites that represented all inhabited continents and wide ranges of human development (Australia, Brazil, Canada, China, Colombia, Finland, India, Kenya, Portugal, South Africa, United Kingdom, and United States). Three moon phases were used in this analysis: full moon (±4 days; reference), half moon (±5–9 days), and new moon (±10–14 days) from nearest full moon. Nocturnal sleep duration, moderate-to-vigorous physical activity (MVPA), light-intensity physical activity (LPA), and total sedentary time (SED) were monitored over seven consecutive days using a waist-worn accelerometer worn 24 h a day. Only sleep duration was found to significantly differ between moon phases (~5 min/night shorter during full moon compared to new moon). Differences in MVPA, LPA, and SED between moon phases were negligible and non-significant (<2 min/day difference). There was no difference in the associations between study sites. In conclusion, sleep duration was 1% shorter at full moon compared to new moon, while activity behaviors were not significantly associated with the lunar cycle in this global sample of children. Whether this seemingly minimal difference is clinically meaningful is questionable.

## Introduction

Folklore has associated behaviors of animals and humans, and even werewolves, to moon phases of the lunar cycle. However, there may be some evidence behind the myth. A good example to illustrate lunar related rhythms in the animal world is the phenomenon of the Palolo worms, which reproduce by swarming during the last quarter of the moon (1, 2). In humans, the empirical evidence that the moon exerts an influence on behaviors is weak (3). However, Cajochen et al. (4) reported that around full moon, sleep duration was reduced by 20 min and sleep quality was decreased in 33 adults measured under controlled laboratory conditions using polysomnography. These findings were later supported by those of Smith et al. (5) who reported a reduction of 25 min/night in sleep duration around full moon in 47 healthy adults tested in the laboratory.

By contrast, a recent population-based study of adults from Switzerland (*n* = 2125) provided no evidence of a significant influence of lunar phases on human sleep (6). Sjödin et al. (7) were the only one to study this issue in children and reported that variations in sleep duration and physical activity measured with accelerometry (i.e., a device detecting acceleration) were significantly associated with the lunar cycle. They observed that children (*n* = 795 Danish children) slept significantly longer on average (~3 min/day) around full moon and were less physically active [~4 min/day of moderate-to-vigorous physical activity (MVPA)]. However, the clinical relevance of these seemingly small differences in children is questionable. The authors were not able to explain the small but significant longer sleep duration around full moon but argued that it could be a consequence of poorer sleep quality. Additional empirical evidence on the widely popular belief that the lunar cycle is related to sleep and activity behaviors of children is needed if we want to debunk this myth. This issue may be particularly relevant to children because they are more amenable to behavior change than adults and their sleep needs are greater than adults.

In order to verify if the full moon is associated with sleep and physical activity of children, we used a 12-country study involving 5812 participants and providing 33,710 24-h accelerometer recordings of sleep and activity behaviors. With such a large and diverse sample of children from around the world, the present study is likely to provide robust evidence of any possible link between the lunar cycle and sleep and activity behaviors. Although the available evidence on the topic is conflicting, we hypothesized that sleep duration would be shorter around full moon and children would be less active.

## Materials and Methods

### Setting

The International Study of Childhood Obesity, Lifestyle and the Environment (ISCOLE) is a multinational, cross-sectional study conducted in 12 countries from all major world regions. ISCOLE study sites included Australia, Brazil, Canada, China, Colombia, Finland, India, Kenya, Portugal, South Africa, United Kingdom, and the United States. These countries have been selected because they represent a wide range of economic development (low to high income), Human Development Index (0.509 in Kenya to 0.929 in Australia), and inequality (GINI coefficient of 26.9 in Finland to 63.1 in South Africa) (8). The design and methods have been published in detail elsewhere (8). By design, the samples were not intended to be nationally representative. Rather, the primary sampling frame was schools, which was typically stratified by an indicator of socioeconomic status to maximize variability within sites. A standard protocol was used to collect data across all sites, and all study personnel underwent rigorous training and certification to ensure the quality of collected data (8). The institutional review board at the Pennington Biomedical Research Center (coordinating center) approved the overarching ISCOLE protocol, and the institutional/ethical review boards at each participating institution also approved the local protocol. Written informed consent was obtained from parents/legal guardians, and child assent was obtained as required by the local institutional/ethical review boards. Data collection occurred between September 2011 and December 2013, representing 28 lunar cycles.

### Participants

The current sample comprised 9- to 11-year-old children from the 12 ISCOLE study sites. Based on an *a priori* power calculation analysis (8), the objective was to enroll a sex-balanced sample of at least 500 participants per site. A total of 7372 children participated in ISCOLE. We excluded participants without valid accelerometry (*n* = 1214), parental education (*n* = 247), or body mass index (BMI) *z*-score (*n* = 5). Changes from summer to winter time and from winter to summer time were also excluded from analysis (*n* = 545 observations from 94 participants). The final analytical dataset comprised 5812 participants and 33,710 accelerometer observations. Participants excluded due to missing data did not differ in their descriptive characteristics (except for BMI *z*-scores, which were significantly higher) compared to those included in the current analysis.

### Classification of Moon Phases

For each measurement using accelerometry (*n* = 33,710 observations), we calculated the distance in days to the date of the closest full moon phase using information from a moon phases calendar (http://www.moonconnection.com/moon_phases_calendar.phtml). This difference was subdivided in three lunar phases: full moon (±4 days; reference), half moon (±5–9 days), and new moon (±10–14 days) from nearest full moon. These classifications of lunar phases are identical to those used in recently published papers (4–7).

### Measurement of Sleep and Physical Activity

Nocturnal sleep time, MVPA, and total sedentary time (SED) were all objectively measured using 24-h accelerometry. Children wore an Actigraph GT3X+ accelerometer (ActiGraph LLC, Pensacola, FL, USA) at their waist on an elasticized belt, placed on the right mid-axillary line 24 h/day, for at least 7 days, including two weekend days. To be eligible for this analysis, children had at least 4 days (including at least one weekend day) with a minimum of 10 h of wear time per day. Data were downloaded in 1-s epochs with the low frequency extension filter and were later reintegrated to 15- and 60-s epochs. Nocturnal sleep duration was estimated using 60-s epochs and using a recently validated algorithm for 24-h waist-worn accelerometers (9). This new algorithm captures total sleep time from sleep onset to the end of sleep, including all epochs and wakefulness after onset, and provides more accurate sleep duration estimates than previous algorithms (9, 10). To be eligible for this analysis, children had at least 3 days of valid sleep (sleep duration ≥160 min/night), including at least one weekend night (Friday or Saturday). After exclusion of total sleep time and awake non-wear time (any sequence of ≥20 consecutive minutes of zero activity counts), MVPA was defined as ≥574 counts per 15 s, light-intensity physical activity (LPA) as between 26 and 573 counts per 15 s, and total SED as ≤25 counts per 15 s, consistent with the widely used Evenson cutoffs (11). After testing for normality, MVPA was log-transformed for analysis.

### Covariates

Age, sex, highest parental education, day of measurement, and BMI *z*-score were included as covariates. A total of 594 participants were missing data on household income, so parental education was used instead as a proxy for socioeconomic status. The highest level of parental education was reported by the parent/guardian and two categories were created to facilitate analysis across sites (did not complete high school vs. completed high school or more). Day of measurement (weekday or weekend day) was included as a covariate because sleep and activity behaviors have been shown to differ between weekdays and weekend days (12). Height and body weight were measured according to standardized procedures by trained study staff (8). BMI (kilogram per square meter) was calculated, and BMI *z*-scores were computed according to the World Health Organization’s reference data (13). Biological maturity was also assessed in ISCOLE using the maturity offset method. However, age and weight are included in the maturity offset calculation, creating collinearity issues, and therefore biological maturity was not included as a covariate in our analyses.

### Statistical Analysis

Statistical analyses were conducted using SAS version 9.4 and JMP version 12 (SAS Institute, Cary, NC, USA). Descriptive characteristics of the sample were presented as mean and SD or as proportions. Multilevel linear mixed model analysis (PROC MIXED) was used to examine the associations between moon phases and sleep/activity behaviors (sleep duration, MVPA, LPA, and SED). Based on this model, we estimated sleep/activity behaviors with “full moon” as the reference category compared with “half moon” and “new moon.” Age, sex, highest parental education, day of measurement, and BMI *z*-score were included as covariates. Multilevel modeling analyses were used to properly account for the hierarchical nature of data (four levels; days nested within individuals nested within schools nested within sites). Study sites were considered to have fixed effects, and schools and individuals were viewed as having random effects. Denominator degrees of freedom for statistical tests pertaining to fixed effects were calculated using the Kenward and Roger approximation method (14). Differences across sites in the associations were examined with the use of interaction terms; site by moon phases interactions were retained when *P* < 0.05. The level of statistical significance was *P* < 0.05.

## Results

**Figure 1:**
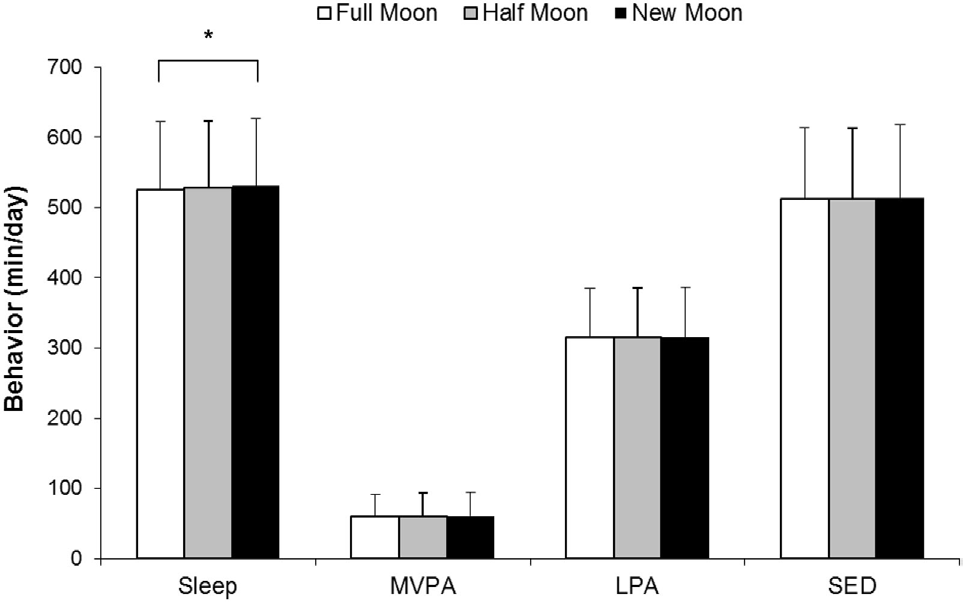
**Sleep and activity behaviors according to moon phases**. Data are presented as mean values and SDs. A multilevel linear mixed model was used with participants and schools as random effects and sites as fixed effects. The model was adjusted for age, sex, highest parental education, day of measurement, and body mass index *z*-score. Full moon (±4 days; includes 30% of observations), half moon (±5–9 days from full moon; includes 33% of observations), and new moon (±10–14 days from full moon; includes 37% of observations). *Sleep duration is significantly shorter during “full moon” compared to “new moon” (*P* < 0.01). MVPA, moderate-to-vigorous physical activity; LPA, light-intensity physical activity; SED, sedentary time.

Descriptive characteristics of the participants are presented in Table 1. Approximately 33% of the participants were overweight or obese, and 58% had at least one parent who completed high school or more. As shown in Figure 1, only sleep duration was found to significantly differ between moon phases. Differences in MVPA, LPA, and SED between moon phases were negligible and non-significant. Sleep duration was 4.9 min/night shorter (*P* < 0.01) at full moon compared with new moon (Table 2), whereas the other behaviors were not significantly different between moon phases (<2 min/day difference). Sleep efficiency, defined as total sleep episode time divided by sleep period time, was also examined and comparisons were non-significant (data not shown). This is not surprising given the small variability in sleep efficiency data (the average sleep efficiency was 96.2 ± 1.3%). Site by moon phase interactions showed no difference between study sites, and there was no evidence of a difference between boys and girls (data not shown). Exploratory analyses using different definitions of lunar phases (e.g., the exact night of the full moon) provided very similar results and reinforced our findings.

**Table 1:** **Descriptive characteristics of participants (*n* = 5812)**.

Age (years)	10.4 ± 0.6
Sex (%)	
Boys	45.7
Girls	54.3
Body weight category (%)^a^	
Obese	12.4
Overweight	20.5
Normal weight	67.1
Highest parental education (%)	
Did not complete high school	41.9
Completed high school or more	58.1
Sleep duration (min/day)	528 ± 53
Moderate-to-vigorous physical activity (min/day)^b^	60 ± 25
Light-intensity physical activity (min/day)	315 ± 53
Sedentary time (min/day)	513 ± 69

*Data are shown as mean ± SD unless otherwise indicated*.

*^a^Body weight status was defined using the World Health Organization criteria (13)*.

*^b^Log-transformed for analysis*.

**Table 2:** **Sleep and activity behaviors according to the moon phases (*n* = 5812 participants and 33,710 observations)**.

Behaviors	Full moon^a^–half moon^b^ **β** (95% CI)	Full moon^a^–new moon^c^ **β** (95% CI)
Sleep duration (min/day)	−2.2 (−4.9; 0.5)	−4.9 (−2.2; −7.6)*
MVPA (min/day)	−0.6 (−2.4; 1.2)	−0.9 (−2.1; 0.5)
LPA (min/day)	−0.5 (−2.1; 1.6)	0.3 (−2.0; 2.0)
SED (min/day)	1.1 (−1.8; 4.1)	−0.9 (−3.7; 1.9)

*Data are presented as unstandardized β coefficients with 95% confidence intervals using a multilevel linear mixed model with participants and schools as random effects and sites as fixed effects. Age, sex, highest parental education, day of measurement, and body mass index *z*-score were included as covariates. Data for MVPA have been back-transformed to their original units*.

*^a^Full moon ±4 days (includes 30% of observations)*.

*^b^Half moon refers to ±5–9 days from full moon (includes 33% of observations)*.

*^c^New moon refers to ±10–14 days from full moon (includes 37% of observations)*.

*Significant difference from full moon (*P* < 0.01).

*MVPA, moderate-to-vigorous physical activity; LPA, light-intensity physical activity; SED, sedentary time*.

## Discussion

The present study was the first to examine the associations between moon phases and sleep and activity behaviors in children across five major geographic regions of the world (Europe, Africa, the Americas, South-East Asia, and the Western Pacific) representing a wide range of sociocultural variability. Findings from this study revealed that sleep duration was, on average, 1% shorter at full moon compared with new moon, whereas other activity behaviors were not different. This finding is consistent with previous observations in adults measured under controlled laboratory conditions (4, 5); however, the magnitude of this difference is much smaller in the present study (~5 vs. 20–25 min). This 5-min shorter sleep duration during full moon compared to new moon is consistent with the finding by Haba-Rubio et al. (6). However, this difference was only statistically significant in the present study because of the greater power (33,710 time-points in the current study vs. 2125 for Haba-Rubio et al.). Our finding is, however, in contrast to what Sjödin et al. (7) recently reported in children, i.e., that children slept 4.1 min longer at full moon compared to new moon in a sample of Danish children aged 8–11 years. Other scientists have also found no influence of the lunar cycle on human sleep in small samples of adults (15–17). Collectively, the current study provides solid evidence to the effect that the associations between moon phases and children’s sleep duration/activity behaviors are not meaningful from a public health standpoint (small effect sizes). Given our large sample size, finding a statistically significant shorter sleep duration around full moon is not surprising. From a clinical standpoint, the magnitude of this effect is unlikely to be important and other aspects of sleep (e.g., sleep quality and timing) also deserve attention.

Mechanisms that may explain the apparent shorter sleep duration at full moon are speculative. The brightness of the moonlight may be one possibility, especially if the window curtain is not sufficiently opaque. At full moon, the illuminance (~0.25 lux) is ~25 times greater than at half moon and 250 times greater than a moonless clear night sky. However, the abundance of artificial light in modern societies where most of us spend evenings and nights indoors suggests that this explanation is unlikely to be valid. It is also plausible that an as yet unidentified circalunar clock exists in humans, such as has been demonstrated in marine worms (4, 5). Evidence that the lunar cycle can modulate sleep in humans can be best demonstrated when measured under highly controlled conditions (e.g., light, temperature, magnetic fields, and hormonal status) of a circadian laboratory study protocol without time cues. Larger reductions in sleep duration in the study by Cajochen et al. (4) can be explained by the *ad libitum* sleep protocol without external time cues, while children in our study were likely woken up at a fixed time on weekdays to attend school. Considering that full moons can occur during school days, this is a limitation to keep in mind that may possibly limit the strength of associations in children.

An important strength of the present study is the large multinational sample of children from low to high income countries across all inhabited continents. We also used objective measurements for sleep and activity behaviors, a highly standardized measurement protocol, and a rigorous quality control program to ensure consistent data collection across all study sites (8). We also used >33,000 days and nights of objectively measured behavior across 28 lunar cycles (~29.5 days each). However, our results have limitations that warrant discussion. First, cross-sectional data are limited in their ability to show causality. Second, accelerometers may be limited in their ability to distinguish between sleeping and waking (they are based on movement detection) and do not provide information on sleep architecture. Moreover, waist-worn accelerometers generally tend to overestimate sleep duration and sleep efficiency compared to devices worn on the wrist (18). However, using only one device to assess both sleep and activity behaviors certainly provides advantages (e.g., less cumbersome for children) and has been reported to give valid estimates of sleep-related behaviors in epidemiology (18). Third, children were not assessed through a whole lunar cycle, but only a week. Although this approach has been used previously (7), it may influence the comparisons reported. Finally, there is always the possibility of residual confounding by unmeasured variables in observational studies.

## Conclusion

The present study provides evidence that nocturnal sleep duration is ~5 min shorter around full moon compared to new moon in this sample of children selected from around the world. However, whether this difference is clinically meaningful is questionable. Future experimental work is needed to determine if the human biology is in any way synchronized with the lunar cycle. Future research should also examine if the full moon may have a larger influence on subgroups of vulnerable children, e.g., those with mental disorders or physical ailments. Whether there is science behind the myth or not, the moon mystery will continue to fascinate civilizations in the years to come.

## Author Contributions

J-PC, MW, AL, MH, KM, PK, MT, TB, SB, MF, GH, RK, AK, EL, CM, JM, VM, TO, VO, OS, MS, CT-L, PZ, and AS designed the study; J-PC and AS analyzed and interpreted the data; J-PC drafted the manuscript; J-PC, MW, AL, MH, KM, PK, MT, TB, SB, MF, GH, RK, AK, EL, CM, JM, VM, TO, VO, OS, MS, CT-L, PZ, and AS critically revised the manuscript for important intellectual content and approved the final version to be published.

## Conflict of Interest Statement

The authors declare that the research was conducted in the absence of any commercial or financial relationship that could be construed as a potential conflict of interest.

## Abbreviations

BMI, body mass index; ISCOLE, International Study of Childhood Obesity, Lifestyle and the Environment; LPA, light-intensity physical activity; MVPA, moderate-to-vigorous physical activity; SED, sedentary time.
